# Four guiding principles for effective trainee-led STEM community engagement through high school outreach

**DOI:** 10.1371/journal.pcbi.1011072

**Published:** 2023-05-25

**Authors:** Stefanie Luecke, Allison Schiffman, Apeksha Singh, Helen Huang, Barbara Shannon, Catera L. Wilder

**Affiliations:** 1 Institute for Quantitative and Computational Biosciences, University of California, Los Angeles, Los Angeles, California, United States of America; 2 Department of Microbiology, Immunology, and Molecular Genetics, University of California, Los Angeles, Los Angeles, California, United States of America; 3 Synergy Quantum Academy, Los Angeles, California, United States of America; SIB Swiss Institute of Bioinformatics, SWITZERLAND

## Abstract

To address ongoing academic achievement gap, there is a need for more school-university partnerships promoting early access to STEM education. During summer 2020, members of our institute initiated QBio-EDGE (Quantitative Biology—Empowering Diversity and Growth in Education), an outreach program for high schools in Los Angeles. In the hope of contributing to increasing diversity in academia, QBio-EDGE aims to make STEM education more accessible for students from historically excluded communities by exposing them to scientific research and diverse scientist role models. This program is led by early career researchers (ECRs), i.e., undergraduate, graduate, and postdoctoral researchers. In our first year, the outreach activities took place during virtual learning, presenting challenges and opportunities within the program development. Here, we provide a practical guide outlining our outreach efforts, key factors we considered in the program development, and hurdles we overcame. Specifically, we describe how we assembled our diverse team, how we established trusting partnerships with participating schools, and how we designed engaging student-centered, problem-based classroom modules on quantitative biology and computational methods applications to understand living systems. We also discuss the importance of increased institutional support. We hope that this may inspire researchers at all career stages to engage with local schools by participating in science outreach, specifically in quantitative and computational fields. We challenge institutions to actively strengthen these efforts.

## The seed that led to “QBio-EDGE”

**Fig 1:**
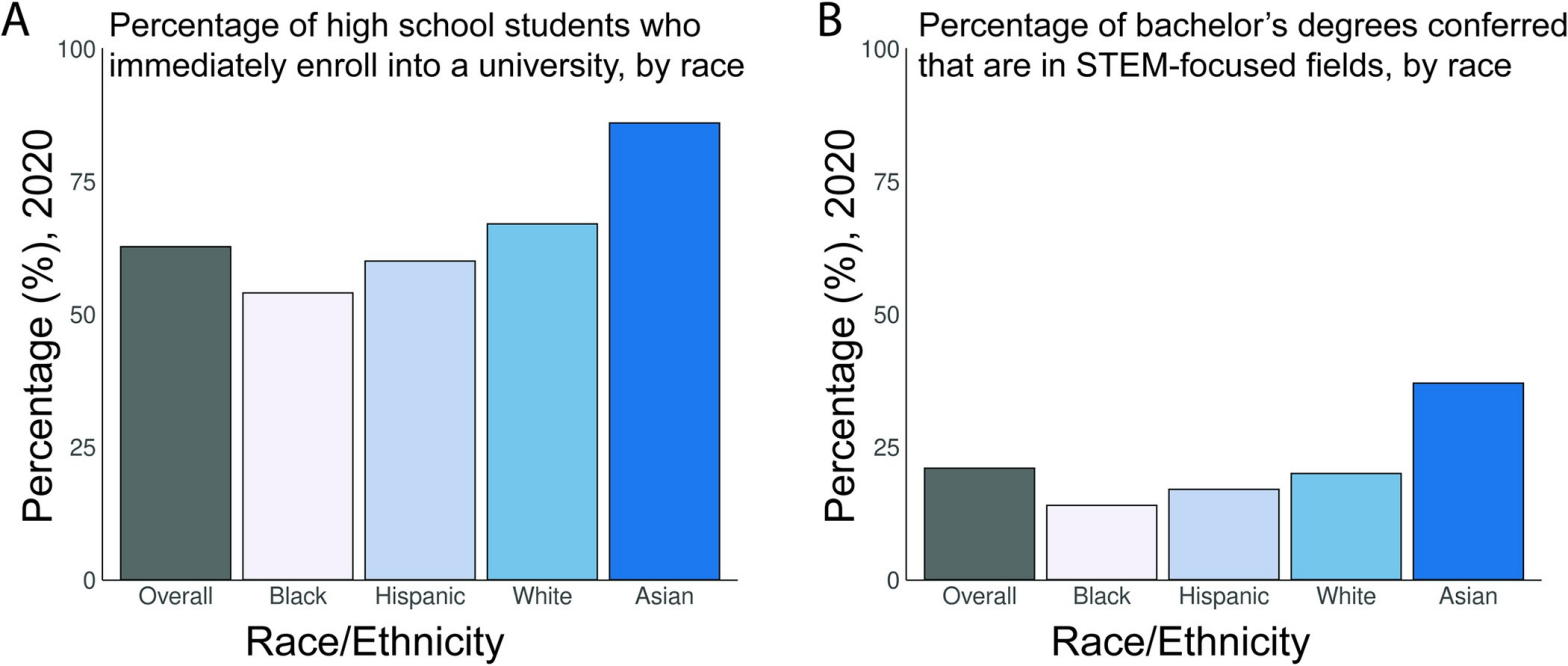
University and college enrollment rates and STEM-focused bachelor’s degrees obtained by race in the US. **(A)** Percentage of high school graduates who immediately enrolled in 2- or 4-year institutions in 2020, by race/ethnicity. Data compiled by [1]. **(B)** Percentage of bachelor’s degree conferred in 2020 that are in STEM-focused fields, by race/ethnicity. Data compiled by [3]. All data were gathered by the US Census Bureau’s Current Population Survey and were estimated for the total population from a 2-year moving average between 2019 and 2020 across a representative sample of households in all US states and D.C. [2].

The achievement gap (e.g., enrollment and degrees conferred) in higher education in the United States remains a major issue. College enrollment for black and Hispanic high school students is less than that of white and Asian students (Fig 1A) and for black students has decreased since 2010 [1,2]. Among degrees earned by black and Hispanic students, a smaller percentage were STEM-focused than for other races (Fig 1B) [2,3]. While the number of math and science courses taken as well as student interest or confidence in STEM during K-12 are good indicators of pursuing a postsecondary STEM degree, these were seen to be less predictive for historically excluded groups [4]. Some factors contributing to racial disparities in postsecondary education are the product of issues that arise during primary and secondary education. These include lack of access to a diverse STEM curriculum, discrimination and implicit bias in the classroom, and lack of racial and ethnic diversity among educators [4–11].

The inclusion of more STEM-based programs in high schools has been shown to improve student interest and success in STEM [4,12–15]. For example, enrollment in an 18-month project-based IT/STEM program improved the critical thinking and reasoning skills of high school students in the program [16]. In addition, educational partnerships between high schools and universities have been shown to increase college enrollment and student interest in future STEM careers [17–19]. Advantages of such partnerships include exposure of high school students to advanced curriculum and establishment of mentor–mentee relationships. Also, implementation of implicit bias training and exposure to racially and ethnically diverse educators and role models has the potential to improve student outcomes [9,11,20,21].

Therefore, there is a need to establish more STEM-focused school-university partnerships and increase exposure of diverse educators and role models to historically excluded groups to improve the disparities in university enrollment and STEM-focused majors [4]. QBio-EDGE (Quantitative Biology—Empowering Diversity and Growth in Education) is an early career researcher (ECR)-run initiative committed to promoting racial and ethnic inclusion in academia by addressing the limited access black and Hispanic students have to public universities. It was formed in response to the murder of George Floyd in May 2020 and the subsequent racial reckoning that increased awareness on issues of diversity and equity.

Here, we identify key factors necessary for a science outreach program in partnership with a high school that is beneficial to the personal and professional development of all parties. We also discuss the challenges in developing an ECR-run program with limited resources. We offer practical guidance and hope that these examples may inspire researchers of all levels to take initiative in science outreach.

## What is QBio-EDGE?

QBio-EDGE is a community-focused organization developed and run by ECRs (undergraduate, graduate, and postdoctoral researchers) at UCLA who partner with local high schools for science outreach. The goal of the initiative is to improve access to STEM education for students in under-resourced communities in the hope of contributing to increasing diversity in STEM-related careers and academia down the line. This is done by providing examples of real-life, practical applications of research skills along with exposure to diverse scientist role models [9,12,13,22–25]. We strive for an authentic, long-term, and reciprocal relationship with participating schools, in which we motivate and empower the students as they approach college applications, while we learn from people of different backgrounds that make up our racially diverse community. An additional function of this initiative is to contribute to our training as ECRs through improving science communication skills and instilling a community-oriented mindset. These activities challenge us to communicate scientific techniques to a broad audience and design engaging classroom activities using remote and in class teaching. We will continue to use these teaching, mentoring, and community involvement skillsets at later career stages, which gives us the potential to change the diversity landscape of academia [25–29].

**Fig 2:**
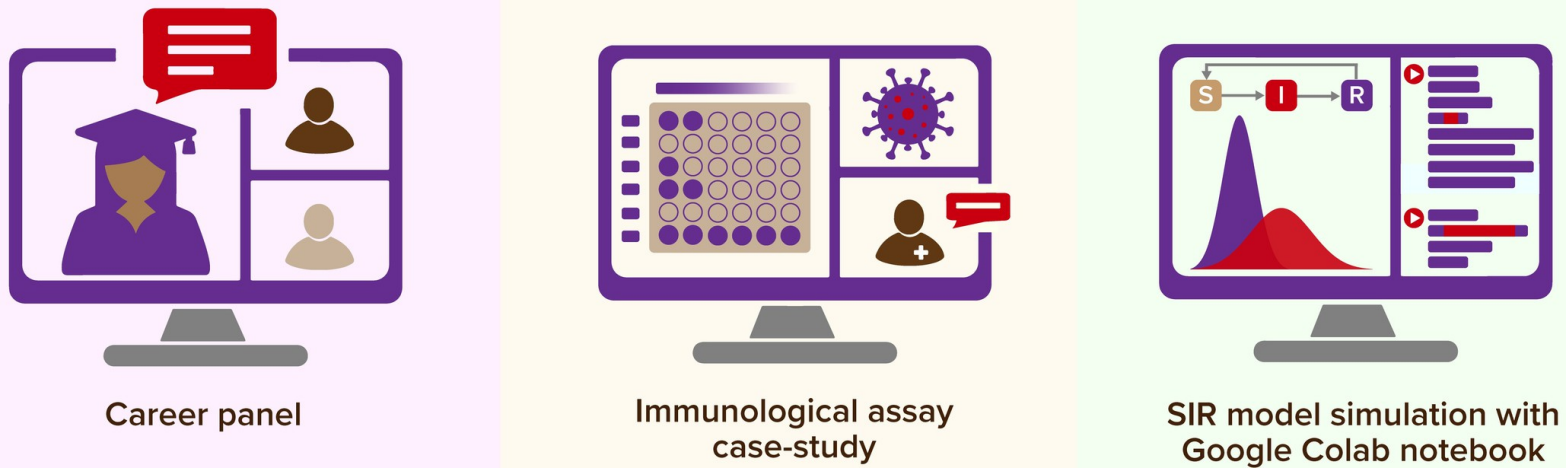
Schematic of the 3 QBio-EDGE sessions. We hosted a career panel, where students asked researchers questions about working in science **(left)**, a Virology session, with a case study where students identified a viral infection through immunological assays **(middle)**, and a Computational Modeling session, where students modified an SIR model of disease spread to answer public health policy questions **(right)**.

In our first year, we established a partnership with Synergy Quantum Academy in South Central Los Angeles, a nonprofit tuition-free charter high school with lottery-based admission and a mission of improving access to STEM education to students in the surrounding area. In 2021, 99.1% of students at this high school were Hispanic and 99.6% of students were socioeconomically disadvantaged [30]. Due to the SARS-CoV-2 pandemic, all sessions were implemented virtually in the program’s first year. We focused on exposure to quantitative and computational biology principles. We worked with students of the 11th and 12th grade “Principles of Biomedical Science” course, taught by Dr. Barbara Shannon, then the school’s Director of STEM education and coauthor of this commentary. Since we specialize in systems immunology, the outreach consisted of 3 sessions over the course of a school year (October 2020, February, and March 2021) (Table 1), each 2 h long: a career panel, a session on immunological assays for virus detection, and an epidemiological modeling session (Fig 2).

**Table 1:** Example schedule for implementation of a new high school science outreach program.

Activity	Jun	Jul	Aug	Sep	Oct	Nov	Dec	Jan	Feb	Mar	Apr	May
**Set up outreach program**												
Recruit outreach program members												
Identify core members												
Initial ideas for outreach: What can we offer?												
Feedback on initial ideas from mentors (e.g., PIs)												
Research potential partner schools												
Recruit members among new campus community												
Design training for outreach program members												
Host training for program members												
**Contact school**												
Initial contact with school(s)												
Finalize choice of partner school(s)												
Dialogue w. school: Which course to contribute to?												
Paperwork required to work with school, if any												
Continued dialogue with teacher about curriculum												
Dialogue with teacher: Define outreach curriculum												
Dialogue w. teacher: Set dates for outreach activities												
Coordinate activities with PIs and admin as needed												
**Workshop 1**												
Design content, materials, and schedule for												
Finalize content, materials, and schedule for												
Host Workshop 1												
Receive student and teacher feedback												
**Participate in college prep event(s)**												
**Workshop 2**												
Design content, materials, and schedule for												
Finalize content, materials, and schedule for												
Host Workshop 2												
Receive student and teacher feedback												
**Workshop 3**												
Design content, materials, and schedule for												
Finalize content, materials, and schedule for												
Host Workshop 3												
Receive student and teacher feedback												
**Plan for following year**												
Dialogue with teacher: Debrief												
Dialogue with teacher: Plans for following year												
Develop membership strategy for following year												
Recruit new members												
Research potential partner schools for following year												

In the career panel, we provided an overview of the diversity of STEM educational and career paths. We asked the students to reflect on their own career aspirations and held dialogues based on students’ fields of interest. In the virology session, the students learned about virus families and life cycles, held presentations on specific viruses, and explored laboratory methods for detecting viruses. In the epidemiological modeling session, the students used mathematical modeling to predict and understand viral spread while learning about advantages and limitations of these model simulations.

Due to the positive student feedback in the first year, we again worked with Synergy Quantum Academy during the 2021 to 2022 school year, organizing the career panel and, based on the course curriculum need and interest of Dr. Shannon, a new 2-day scientific module with a bioinformatic focus on genome-wide association studies (GWAS). We also participated in a college application essay workshop with Synergy students and initiated a multi-year one-on-one mentoring program between UCLA ECRs and Synergy students, whom we hosted in a series of lab workshops and university tour. To expand our program, we connected with a privately run high school in South LA, with students from the surrounding economically disadvantaged neighborhood who are predominately Hispanic or black. There, we implemented the new GWAS module with the students in their Biology course. All 2021 to 2022 events were in person or hybrid.

We hope that our outreach efforts at both high schools mark the beginning of a continued relationship with these schools and students that provides guidance about college applications, college life, and academic and STEM careers as well as guidance on the community needs and ways QBio-EDGE can engage with community members.

## Steps to create a successful ECR-run outreach program

### Step 1: Assemble a diverse and motivated team

For successful events, a team of committed organizers is essential. These groups can form from within a research group or a larger research community. We started with a team of organizers from 1 research group and recruited researchers from the larger UCLA community, especially from the Institute for Quantitative and Computational Biosciences (QCBio), by advertising at QCBio community and graduate program events. Our committee consisted of a core group of 4 to 6 organizers who met weekly throughout the year with app. 10 additional members joining in the weeks leading up to and on the event days. There were roughly equal numbers of graduate students and postdoctoral researchers and a few undergraduate researchers in both the core group and the larger team. This provided a useful balance of perspectives and experiences. Members participated in anywhere from one to all events, based on availability, expertise, and skill sets. Members chose tasks of interest to them, and roles were not restricted by career stage in any way. Leadership of meeting facilitation rotated. With this flexible setup, we were able to divide the preparatory workload, such that we could develop well-planned activities without overwhelming organizers. The many educators (7 to 8 for the science workshops, 14 for the career panel) also enabled small facilitator-to-student ratios (1:4 to 1:2) for all breakout groups during the sessions, which was especially critical for student engagement in the virtual environment. Although significant interest, passion, and time commitment from the organizers was essential, especially when developing an independent outreach program from scratch, our committee structure allowed for flexibility in the members’ commitments over time, which we found to be important for maintaining enthusiasm in the voluntary outreach work. We anticipate that this distribution of responsibilities and broad recruitment effort will aid the longevity of QBio-EDGE. Yearly changes in membership are anticipated in a university setting, so regular recruitment of new members is important. Three out of app. 17 ECRs involved at some point in the first year of programming participated regularly in the second year, and we recruited 5 new organizers. In addition, app. 8 other ECRs (either with first year experience or newly recruited) contributed to individual events.

Additionally, it is important to assemble a team that is diverse in academic experiences and personal identities. Lack of inclusion and isolation is often a key factor in students and researchers dropping out of college and academic career paths [31–33]. As researchers of different genders, races (e.g., black, white, Asian), ethnicities (e.g., Hispanic), and nationalities (e.g., US, Germany, France, China, India) as well as educational backgrounds, scientific fields, and career stages, QBio-EDGE facilitators could physically demonstrate to students the diversity of people who work in academia to offset some of these sentiments. Providing visibility through personal identity-matched role models can improve academic interest, performance, and retention [24]. Practically, it also allowed us to cater more specifically to student interests. For example, during the career panel session, we facilitated breakout groups for different areas of science (e.g., biology, engineering/physics, math/computer-science, health sciences), each staffed with multiple researchers at different career stages (ranging from undergraduate student to assistant professor) with varying personal identities.

### Step 2: Connect with local schools to form an equitable and trusting partnership

Outreach should always address the expressed needs and goals of the community it aims to serve. Identifying a school that is suitable for and open to the outreach activity can often be a first hurdle, especially when researchers are new to the community. To make a meaningful contribution, we partner with schools that serve students who have either historically been excluded from college campuses and/or that have little STEM-focused resources. As a new, ECR-run organization without established contacts, we leveraged a connection to Synergy Quantum Academy, which was founded in 2011 to close the achievement gap in South Central Los Angeles. It mainly serves students of color from socioeconomically disadvantaged backgrounds and strives to improve STEM access, sharing the goals of our initiative. One of our team members is an alumna of the school and described how despite the school’s STEM focus, she would have benefited from more training in research skills to facilitate participation in academic research during college and from exposure to role models working in STEM-focused careers.

This alumna initiated the first contact with the school during the summer (Table 1) and helped form a trusting partnership. In absence of such personal contacts, we recommend researching local schools (local undergraduate students can be particularly helpful) and reaching out to their leadership teams (e.g., principals or program directors) directly. Researchers in California, for example, can use the California school dashboard [30] to identify nearby schools that serve predominantly socioeconomically disadvantaged students. Another option is to network with outreach and college recruitment programs on campus to build on existing relationships.

We found it important to communicate with school teachers prior to any activities to educate ourselves about the students’ needs, so they can directly inform planning [34]. We met with Dr. Shannon before the start of the school year (Table 1) to learn more about how we could best serve her students. She expressed the importance of high schools and universities having an ongoing dialogue regarding required skill sets to provide the students with the best preparation possible, especially for STEM courses at the college level. We followed her suggestion to integrate our workshops in her “Principles of Biomedical Science” course due to the best match in topic. This increased the chances of our workshops being meaningful to the attending students, since they enrolled in the course based on interest. At Synergy Quantum Academy, 70% of students on the Biomedical Science track have taken courses involving some computational skills, but after discussions with Dr. Shannon, it was clear that there remained a particular need to increase exposure of students to examples of the application of computational skills to real-world problems outside of computer science classes. We coordinated the topics, timings, and length of our outreach sessions to best integrate them into the existing course curriculum and class times. By coordinating our sessions with the teacher, we facilitated students’ learning by connecting our sessions with their prior knowledge [35].

Maintaining open and regular communication throughout the year in a process of co-creation with both teacher and students, applying principle of empathetic teaching and student-centered learning [28], allowed us to improve the utility of our sessions. For example, when we provided the students time to ask questions during the career panel, we observed a desire to know more about attending college and majoring in science compared to information about later career stages. Following these observations, we redesigned the career panel for the following year to include more information about undergraduate science education and recruited more current undergraduate students to share their experiences and expertise. Additionally, after feedback from the teacher, we learned to leave more time for the students to respond to questions posed to the class in future sessions.

One goal was to establish a more authentic, long-term relationship with the school and the students. We accomplished this by spreading the outreach sessions over the school year (October, February, and March) with the same set of students (19 of the 24 students of “Principles of Biomedical Science” course participated in the set of activities), participating in a college essay writing event held by the school in the following October, and adding a mentoring program in the following school year (for a new cohort of students).

Another aspect that facilitated respectful interactions with students was preparatory training. Our facilitators completed a six-module online course on implicit bias management, which is recommended and provided by the University of California [36], and participated in an inclusive classroom training focused on affirming students’ identities [37]. Additionally, the team member who had attended this high school shared her experiences with us and provided background information on the history of systemic racism and its effect on communities in Los Angeles. These activities were intended to improve awareness of these issues in our facilitators, many of whom were unaware of the local and US-specific circumstances due to their international background, and to provide them with strategies for thoughtful and intentional interactions with the students. Through this kind of intentionality, the development and execution of outreach efforts can remain a collaborative process with the communities they serve.

### Step 3: Design engaging, learner-centered classroom activities to promote scientific literacy

A shared goal between us and the course instructor was incorporating quantitative biology and its application to real-life problems into the course curriculum. Therefore, we suggested 2 topics: assaying an infection (virology) and predicting the course of a pandemic (computational modeling). After coordinating the topics with the class teacher, we set out to design classroom activities with original teaching materials.

As researchers with limited teaching experience, a key challenge in our first year was to create classroom activities with the following requirements: (1) be engaging and enjoyable for the students; (2) provide an interactive research-like experience [27]; (3) communicate scientific principles; and (4) be deliverable virtually through the Zoom video-conferencing application. To meet these requirements, we designed our materials to use methods found to be useful in pedagogy literature.

**Fig 3:**
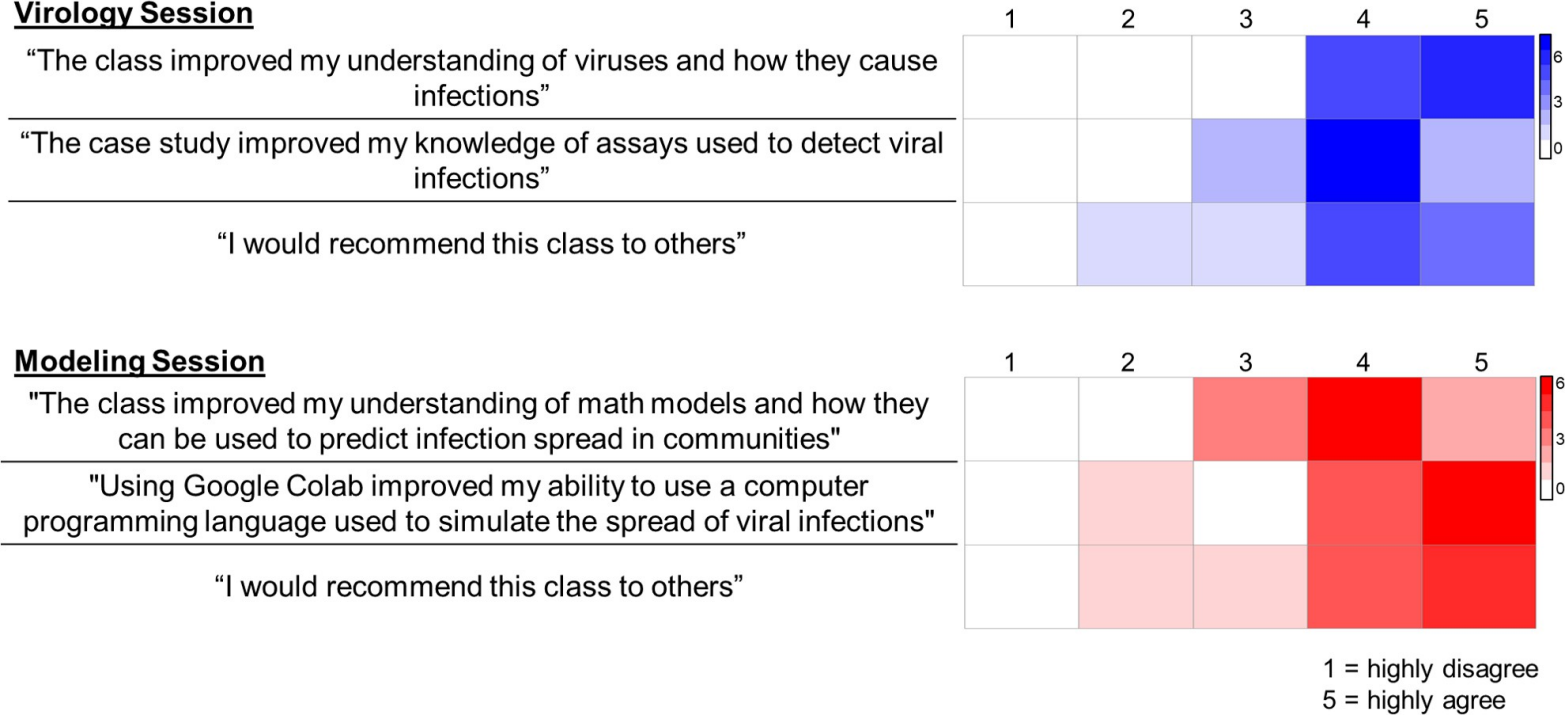
Selection of responses from student feedback survey. Students were asked to rate how much they agreed with the listed statements as a part of a feedback survey following the Virology **(top)** and Epidemiological Modeling **(bottom)** sessions, and 11 out of 19 students responded to each survey.

To promote critical thinking and feelings of empowerment while engaging students, we took a student-centered approach with problem-based learning [27,28,38,39]. This approach was applied to the virology and computational modeling sessions, where the students took on the role of decision makers solving specific problems: doctors diagnosing a viral infection and public health officials making policy recommendations in a pandemic. For the viral infection case study, we prepared handouts explaining the detection assays, which the students worked through in groups, with a facilitator to guide their inquiry [35]. We prepared mock results, which the students interpreted and combined with hints from the patient’s history to determine the infectious agent. In the epidemiology session, each group used a variation of an SIR model to investigate the effect of a different public health intervention on viral spread. The models were coded in advance by the team on Google Colaboratory [40], a free, web-based interactive Python notebook. Prior to the session, we asked the students about their background in calculus and coding and found that most students had limited experience with both, while some had taken calculus or computer science courses. We designed the session to appeal to students at different levels: the students were asked to alter or add parameters to their model and discuss the effects this had on simulations of viral spread. This platform allowed beginners to identify the correct places to change parameters and interpret their effects, while advanced students read the full code to learn about the full structure of the model and how it was implemented into code. This inclusive, active learning approach was well received by students (Fig 3). Additionally, the emphasis on collaboration promoted an interactive experience resembling scientific research.

Based on feedback we received from students using Google Forms, many students (1) would recommend these outreach activities; (2) increased their understanding of the topics discussed; and (3) were able to improve their scientific communication skills (Fig 3). However, the response rate was not very high (app. 58%). For a higher survey response rate, we recommend presenting a link to anonymous pre- and post-session surveys to the class and giving students set time during the session to fill out the survey. Since the de-identified surveys were collected for feedback and assessing the effectiveness of the teaching modules, an IRB approval was not obtained. In addition to the surveys, feedback from Dr. Shannon indicated the students’ appreciation for the real-world science application aspects of the workshops, such as public health policy in a pandemic, which led to enthusiasm for attending the workshops. Through her feedback, we learned the utility of obtaining live feedback from students, such as check-ins on pace and comprehension, a teaching skill we can employ in other settings.

The remote nature of the outreach in its first year reduced the financial resources, time investment, and paperwork required on our side, making it easier to recruit sufficient facilitators and allowing us to focus on the content of the sessions rather than the logistics. It challenged us to make extra efforts to hold the students’ attention, which we attempted to address through problem-based learning and a high facilitator-to-student ratio during the group work (e.g., 1 facilitator and 3 to 4 students). Access to electronic devices and internet connection for participation in the sessions is another challenge for remote programs that the school had already addressed since teaching was entirely remote during that time. Overall, in our subjective experience, a virtual outreach program can work well and may be a useful start, but in-person sessions nonetheless appear better suited to convey scientific excitement and, at least in a local outreach program, provide more equitable access to the program.

### Step 4: Cultivate a supportive environment at home institution to sustain outreach program

A supportive environment and sufficient resources are crucial for designing an effective outreach program and for its continued growth. Thus, we recommend a multitiered approach in which individuals from all academic ranks participate. We urge principal investigators (PIs) and others in leadership positions, such as institute and program directors, to leverage resources that may be more limited to an ECR-only group: funding, access to professional and social connections, and laboratory space for activity use.

While the responsibility of securing funds and resources is not required of PIs, we recommend this collaborative approach to leverage the institutional connections PIs have with intramural and extramural funding programs. Additionally, this collaborative approach has the potential to promote institutional support of science outreach programs with local community partners and fosters University–community relationships. We also recommend that PIs promote the program by encouraging involvement of ECRs and faculty. While senior academics could also contribute a more active leadership role, for our group, an ECR-initiated and -run format allowed us to develop our ideas independently and creatively without pressure and avoided performative participation. For researchers, we recommend finding peers passionate about community involvement. Forming the initial critical mass will ease distribution of responsibilities and new peer connections ensure the sustainability of the program. If there is lack of support from PIs or other leadership, we recommend ECRs engage with their University’s Office of Outreach and Engagement or an equivalent program, as well as other community organizations for access to funding and other resources.

We were fortunate that faculty support of our efforts was provided for the program from the start by faculty members giving advice when needed and providing a platform to present our efforts to the UCLA QCBio Institute, which was helpful in recruiting new members. This support motivated QBio-EDGE members and allowed them to devote time needed for a successful program. Institutional support was also demonstrated when the efforts of QBio-EDGE were acknowledged with the “Initiating and Leading Efforts in Diversity and Equity Award” from QCBio.

Unsolicited commitment from faculty at our institute to help identify funding sources has been motivating and provides flexibility for planning in-person activities. Since the inaugural year was primarily virtual due to the COVID-19 pandemic, not many financial or spatial resources were required. Due to this, no initial solicitations were made to intramural and extramural grant funding programs nor lab spaces sought out for learning modules. Since the inaugural year, university lab space has been used during campus tours for the students in the program, while the learning modules have occurred in the high school classrooms. Financial support for busing students has been provided by the high school. Funds provided by UCLA QCBio were used to purchase food for the students during the campus tours. In the future, financial support will be requested to reimburse ECRs travel to and from the high schools. If institutes and research group leadership truly want to support outreach activities, financial and administrative support is a simple way of showing the seriousness of the institution’s commitment to the cause.

## Conclusions

Our efforts show that grassroots initiatives by ECRs can successfully spark community involvement through scientific outreach. We learned how to communicate scientific principles effectively to high school students and saw that immersive outreach is possible even within a virtual setup. With support from our institution and the class instructor, we were able to prepare didactically useful and engaging teaching materials. With the incorporation of student feedback and critical self-reflection, we are improving our materials for future activities as we expand our reach. We encourage all ECRs to embrace outreach activities and science communication as essential elements of their training. In particular, ECRs preparing for faculty applications can benefit from strengthening their leadership and teaching skills through these endeavors. Researchers will learn firsthand about their community, challenging existing implicit biases and enabling more informed advocacy.

For a broad and lasting impact, however, more institutional support for science outreach is needed. In the future, departments may have outreach programs integrated into their graduate programs. With institutional support, programs such as QBio-EDGE and others [41–46] can successfully weather member turnover and perform more financially intensive activities. Reliance of these initiatives on strictly volunteered effort can be unsustainable and unreasonably demanding of ECR’s, especially those from historically underrepresented groups, which often contribute disproportionately to diversity efforts [47]. Participation in such outreach efforts should be recognized as essential work, and hence should be compensated accordingly [48]. We urge faculty and heads of science departments to prioritize outreach activities as a core mission of academic institutions and encourage all research community members to participate in them. Including some outreach activity as a requirement in PhD programs would train researchers early to regularly engage with their community and alleviate some of the uneven burden of diversity efforts on historically underrepresented groups.

Fostering a mentoring and community-oriented mindset and the related skills early on benefits both the community and ECRs. It also has the potential to change the diversity landscape of academia and other scientific careers if researchers continue efforts towards improving diversity as they move into leadership positions. We are convinced of the value of community outreach and strongly hope this reflection on our experiences can guide other ECRs to develop their own outreach efforts, such that QBio-EDGE will soon become one of many organizations of researchers prioritizing educational outreach to historically excluded and disadvantaged students.

### Access to our materials

Detailed information on our program and all our classroom materials can be found at: https://qcb.ucla.edu/qbio-edge.
